# The Nature of Expertise in Fingerprint Matching: Experts Can Do a Lot with a Little

**DOI:** 10.1371/journal.pone.0114759

**Published:** 2014-12-17

**Authors:** Matthew B. Thompson, Jason M. Tangen

**Affiliations:** School of Psychology, The University of Queensland, St Lucia, Queensland, Australia; University of Akron, United States of America

## Abstract

Expert decision making often seems impressive, even miraculous. People with genuine expertise in a particular domain can perform quickly and accurately, and with little information. In the series of experiments presented here, we manipulate the amount of “information” available to a group of experts whose job it is to identify the source of crime scene fingerprints. In Experiment 1, we reduced the amount of information available to experts by inverting fingerprint pairs and adding visual noise. There was no evidence for an inversion effect—experts were just as accurate for inverted prints as they were for upright prints—but expert performance with artificially noisy prints was impressive. In Experiment 2, we separated matching and nonmatching print pairs in time. Experts were conservative, but they were still able to discriminate pairs of fingerprints that were separated by five-seconds, even though the task was quite different from their everyday experience. In Experiment 3, we separated the print pairs further in time to test the long-term memory of experts compared to novices. Long-term recognition memory for experts and novices was the same, with both performing around chance. In Experiment 4, we presented pairs of fingerprints quickly to experts and novices in a matching task. Experts were more accurate than novices, particularly for similar nonmatching pairs, and experts were generally more accurate when they had more time. It is clear that experts can match prints accurately when there is reduced visual information, reduced opportunity for direct comparison, and reduced time to engage in deliberate reasoning. These findings suggest that non-analytic processing accounts for a substantial portion of the variance in expert fingerprint matching accuracy. Our conclusion is at odds with general wisdom in fingerprint identification practice and formal training, and at odds with the claims and explanations that are offered in court during expert testimony.

## Introduction

Expert decision making often seems impressive, even miraculous. People with genuine expertise in a particular domain can perform quickly and accurately, and with little information. Chess players [1], fire ground commanders [2], radiologists [3], [4], dermatologists [5], and ballplayers [6], for example, are capable of extraordinary feats of categorization. The exemplar theory of categorization posits that people put everyday objects and situations into categories—“That is a chair,” “That is a dog,” “That situation is dangerous”—by comparing the similarity of a novel instance to individual prior instances [7], [8]. The same holds for instances that most of us don't encounter everyday but that experts do—“That is a bad chess move,” “That building is about to collapse,” “That is an abnormal mammogram,” “That is measles,” “That is a curveball.” The nature of this categorization process has been characterized as intuitive, unconscious, associative, and effortless (see [9] for a review), so long as people receive feedback and the environment is sufficiently regular [10], [11].

Understanding where expertise lies in various domains can help us to understand the nature of expertise more generally. Expertise in sports, for example, lies in the anticipating an opponent's body position before a pitch or serve [6]; wine expertise seems to be based in part on the wine connoisseur's linguistic skill [12]; diagnosticians and radiologists can advance tentative hypotheses quickly and effortlessly [3], [13]; and chess masters can remember the exact configuration of pieces on a chessboard after only a few seconds, and can play blindfolded [14]. In the series of experiments presented here, we work to understand where expertise lies for a group of experts whose job it is to identify the source of crime scene fingerprints.

The comparison and identification of crime scene fingerprints is based on human decision making, not a computer algorithm. When a print is lifted from the scene of a crime, it is sent to a professional fingerprint examiner who compares the print to that of a suspect or to the output of a database search. But the ultimate decision about whether the prints came from the same person or two different people is up to the examiner. Fingerprint examiners, with careers often spanning decades, spend several hours a day examining these highly structured fingerprint impressions, which makes them a fascinating expert group for study in their own right. These examiners, however, have testified in court for the past one hundred years as to whether two fingerprints from the same person or different people in the absence of formal data on the extent to which they can correctly match fingerprints to one another [15]–[17]. Furthermore, there are few experiments that have directly investigated how competent these examiners are, how they make decisions, or the factors that affect their performance [18]–[22].

Formal research programs have begun. Tangen, Thompson, and McCarthy [23] and Thompson, Tangen, and McCarthy [22], [24] tested the matching accuracy of qualified, court-practicing fingerprint examiners in a signal detection paradigm, and found that experts were exceedingly accurate compared with novices. Experts tended to err on the side of caution by making more errors of the sort that could allow a guilty person to escape detection than errors of the sort that could falsely incriminate an innocent person. A similar experiment, with examiners from the US Federal Bureau of Investigation, produced similar results [25], [26]. An examiner's expertise seems to lie, not in matching prints *per se*, but in discriminating highly similar but nonmatching prints [22].

Several studies show that the judgments of fingerprint examiners can be affected by domain irrelevant information (e.g., about the suspect, police suspicions, and other aspects of the case; [27]–[29], see [30] for a review]. Busey and Vanderkolk [31] conducted the first experiment aimed at investigating the nature of fingerprint matching expertise, finding behavioral and electrophysiological evidence that experts process prints configurally, and that experts move their eyes differently from novices [32], [33]. Despite these contributions, we still know very little about the performance of experts and the nature of their decision making [34], especially given the history of claimed infallibility [35] and zero error rate for fingerprint comparisons [36]. Our approach to understanding where expertise lies in fingerprint identification specifically, and the nature of expertise generally, is to manipulate the amount of information available to expert and novice participants.

Examiners suggest that careful, deliberate analysis is the basis of the work that they do [33], [37], but a hallmark of genuine expertise is the ability to accurately perform a domain relevant task quickly and effortlessly [38]. The exemplar theory of categorization posits that classification is easy for people who have acquired a large number of exemplars from the various categories, because this experience allows them to categorize new items based on the similarity of the new item to the previously encountered exemplars [39], [40]. Much of diagnostic medicine, for example, is thought to be accounted for by the rapid retrieval of previous instances—non-analytic processing [41]. If fingerprint examiners likewise draw on a repository of prior instances when making judgments about new prints, then they should also be able to perform accurately even when the amount of information available in the prints is significantly reduced.

In the four experiments that follow, we manipulate the amount of “information” available to novice and expert fingerprint examiners. In Experiment 1, we limit information by adding visual noise to fingerprint images and present them either upside down or right-side up. In Experiment 2, we limit information by spacing the fingerprint pair in time by a few seconds. In Experiment 3, we space the prints even further apart in time to test people's long-term memory for the patterns. In Experiment 4, we limit information by flashing the pair on screen for only 2 seconds before asking participants whether they match or not. These experiments will help to reveal how fingerprint examiners process matching and non-matching fingerprint pairs by limiting the amount of information available.

## Experiment 1: Inversion in Noise

Faces are far more difficult to recognize when they are presented upside down compared to right-side up (see [42] for a review). This “inversion effect” might seem counterintuitive since the visual information in an inverted face is identical to an upright face, and the relationship between the facial features is the same. But people have far more experience with upright faces compared to inverted faces, and this asymmetry in recognition performance can been chalked up to the asymmetry in one's experience with the materials [43]. People are experts with upright faces, but novices with inverted faces. The inversion effect may therefore be regarded as an index of experience that one has accumulated with a particular class of stimuli [44].

Several recent experiments have demonstrated large expertise effects in fingerprint matching [22], [23], [26], and experience appears to play an important role [24]. Experts also have a lot of experience with upright prints, and almost always reorient them before making a comparison [31]. We therefore expect that expert fingerprint examiners will perform well when matching upright prints, but not when matching inverted prints. Novices, on the other hand, should perform the same on both upright and inverted prints. Our hypothesis is based in part on a related experiment by Busey and Vanderkolk [31], who provided electrophysiological evidence that experts process inverted fingerprints differently than novices, and also found that experts performed better than novices at identifying fragments of fingerprints presented in visual noise after a short delay.

In Experiment 1, we present novice and expert fingerprint examiners pairs of fully rolled fingerprints that were either upright or inverted, and asked them to indicate whether the prints match or not. Artificial noise was added to each image to obscure some of the visual features in the fingerprints making the identification task more difficult for examiners who generally perform such tasks almost perfectly [23]. We expect that experts will show a greater performance difference on upright versus inverted fingerprints compared to novices.

### Method

#### Ethics Statement

This experiment, and the three that follow, were approved by The University of Queensland Behavioural & Social Sciences Ethical Review Committee (2010000106), and all participants gave written informed consent.

#### Participants

Novices were 30 undergraduates from The University of Queensland who participated for course credit and who had no experience with identifying fingerprints. Experts were 13 qualified, court-practicing fingerprint experts with an average 13.5 years (*SD* = 8.2) experience from four Australian police organizations: The Australian Federal, New South Wales, Victoria, and Queensland Police.

#### Procedure

The experiment was a mixed 2×3×2 design: 2 (Expertise: expert, novice; between subjects) ×3 (Trial: target, similar, random; within subjects) ×2 (Orientation: upright, inverted; within subjects). We presented participants with a pair of prints displayed side-by-side on a computer screen. The prints appeared onscreen for 60 seconds and participants were then asked to judge whether the two prints were the same or different, using a confidence rating scale ranging from 1 (sure different) to 12 (sure same). Judgments were reported by moving a scrollbar to the left (“different”) or right (“same”). The scale forced a “match” or “no match” decision, where ratings of 1 through 6 indicated “no match,” whereas ratings of 7 through 12 indicated a “match.” Half of the prints in the set were presented upright and half were inverted. For each participant, a total of 36 pairs of prints were randomly allocated to the Orientation condition (with the restriction that 18 were upright and 18 were inverted) and to Trial condition (with the restriction that 12 were target pairs, 12 were similar pairs, and 12 were random pairs). The order of presentation for Trial condition was random, but Orientation was counterbalanced such that half the participants saw the first half of the set of prints inverted and the second half upright, and the other half of participants saw the first half of the set of prints upright and the second half inverted.

#### Stimuli

**Figure 1:**
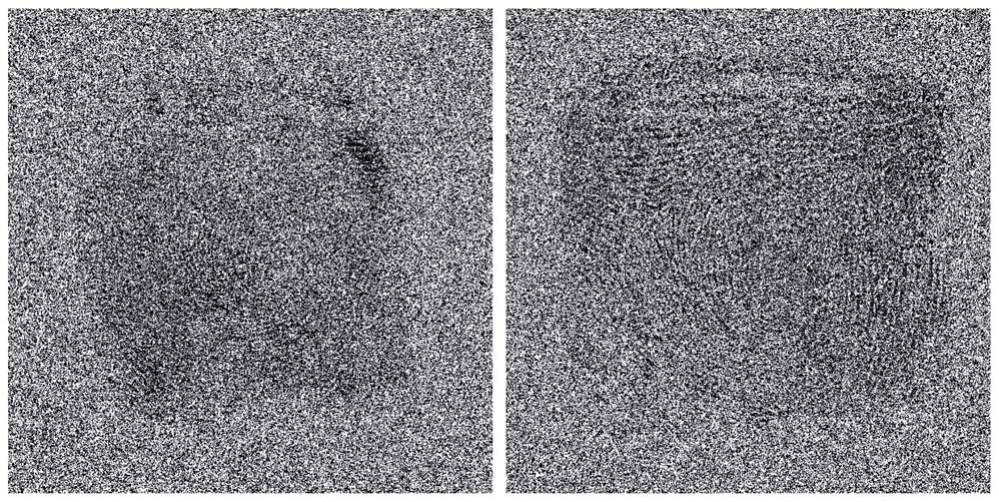
An example of a pair of inverted fingerprints with artificial noise. The print on the left is the ‘crime scene’ print and the print on the right is a similar non matching ‘suspect’ print.

All prints were individual, ‘fully-rolled’ exemplar prints that were scanned and extracted from 10-print cards. On each trial, two images appeared on the screen as illustrated in Fig. 1. There were three types of trials: (1) matches, where the two images were prints of the same finger from the same person, and were separate instances; (2) similar nonmatches, where the two images were prints from two different people, but were deemed similar by a database search algorithm; and (3) nonsimilar nonmatches, where the two images were prints from two different people, and the print on the right was randomly sampled from the set of targets. Each print on the left acted as one of the three trial types across the experiment.

There were 36 left side prints in total, and each was paired with a matching print (i.e., a new instance of a print from the same finger of the same person) to create a match trial, or a similar nonmatching print (the result of a national database search as described below) to create a similar nonmatch trial, or a nonsimilar nonmatching print (the right side print was randomly selected from the set of new instance target images) to create a nonsimlar nonmatching trial. For each participant, each simulated print was randomly allocated to one of the three trial types, with the constraint that there were 12 prints in each condition. So the total number of possible pairs across the experiment was 36×3 = 108, but each participant responded to only 36 trials in total. In addition to the three trial types, each trial could be presented either upright or inverted. The two fingerprint images on each trial were always either both upright or both inverted. Each participant saw 12 matching pairs (6 upright and 6 inverted), 12 similar nonmatching pairs (6 upright and 6 inverted), and 12 nonsimilar nonmatching pairs (6 upright and 6 inverted). Allocation to either upright or inverted was random, but was counterbalanced by having either all upright prints presented first or all inverted prints presented first for each participant.

The matching and nonsimilar nonmatching prints were sourced from the Forensic Informatics Biometric Repository (FIB-R.com), and similar nonmatching prints were obtained by searching the Australian National Automated Fingerprint Identification System. Artificial noise was added to each of the fingerprint images using the “Speckle” function from the Image Toolbox in Matlab. “Speckle” is multiplicative noise algorithm based on the equation *J* = *I*+*n*×*I*, where *I* is the image and *n* is uniformly distributed random noise with mean 0 and variance *v*. The value for *v* was set to 10 for each image. The amount of visual noise was based on the judgments from three qualified fingerprint experts who were asked to indicate when they thought there was no longer enough detail to make an identification.

### Results

**Figure 2:**
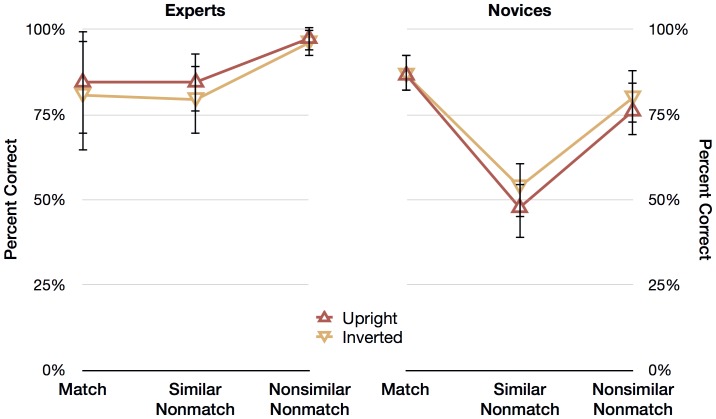
Results from Experiment 1: Inversion in Noise. Experts' and novices' mean percentage of correct responses for the three trial types (match, similar nonmatch, and nonsimilar nonmatch) and the two orientations (upright and inverted). Error bars represent 95% within-cell confidence intervals.

Fig. 2 shows the mean percentage of correct responses of experts (on the left panel) and novices (on the right panel) for the two fingerprint orientations (upright and inverted) and the three trial types (match, similar nonmatch, and nonsimilar nonmatch). We subjected the percentages of correct responses to a 2 (Expertise: experts, novices) ×2 (Orientation: upright, inverted) ×3 (Trial: match, similar nonmatch, nonsimilar nonmatch) mixed analysis of variance (ANOVA). The main effects and interactions that follow are averages collapsed across the twelve experimental conditions, so the percentages described in-text cannot be mapped directly to those depicted in Fig. 2.

#### Main effects

Collapsing across Orientation and Trial, experts were more accurate (87.2% correct) than novices (71.9% correct), resulting in a significant main effect of Expertise, *F*(1, 41)  = 20.869, *p*<.001. Collapsing across Expertise and Trial, there was no difference between upright (79.5%) and inverted prints (79.5%), and so there was no main effect of Orientation, *F*(1, 41)  = 0.001, *p* = .983, *ns*. Collapsing across Orientation and Expertise, although not of particular interest, there was a significant main effect of Trial, *F*(2, 82)  = 15.320, *p*<.001, and follow-up contrasts revealed that nonsimilar nonmatch trials (87.4%) were not significantly different from match trials (84.7%), *F*(1, 41)  = 0.406, *p* = .528, *ns*, but similar nonmatch trials (66.4%) were significantly different from match trials (87.4%), *F*(1, 41)  = 14.621, *p*<.001.

#### Interactions

Of particular interest was the relationship between Orientation and Expertise, because we expected that novices would be just as accurate with inverted prints as with upright prints, but that experts would be less accurate with inverted prints than upright prints. But, as is clear from Fig. 2, there was no significant interaction between Orientation and Expertise, *F*(1, 41)  = 2.799, *p* = .102, *ns.* That is, unexpectedly, experts were no more affected by inversion than novices were. As expected, there was a significant interaction between Trial and Expertise, *F*(2, 82)  = 9.374, *p*<.001, and follow-up comparisons reveal that the increased accuracy for nonsimilar nonmatch trials, compared to match trials, was greater for experts than for novices, *F*(1, 41)  = 6.946, *p* = .012. Furthermore, the decrease in accuracy for similar nonmatch trials compared to match trials was greater for novices than for experts, *F*(1, 41)  = 13.612, *p* = .001. Finally, there was no significant interaction between Orientation and Trial, *F*(2, 82)  = .305, *p* = .738, *ns*, and no significant three-way interaction between Orientation, Expertise, and Trial, *F*(2, 82)  = .421, *p* = .658, *ns*.

### Discussion

In Experiment 1, we tested whether fingerprint experts or novices would show an inversion effect during a matching task. We expected that novices would be just as accurate at matching fingerprints when the prints were inverted or upright, but that qualified experts would be less accurate for inverted than for upright prints. We did not, however, find any evidence for an inversion effect; experts and novices were both just as accurate for inverted prints as for upright prints. This lack of effect is in contrast to Busey and Vanderkolk [31] who provided electrophysiological evidence for an expertise effect in the form of a delayed N170 component for experts in response to inverted fingerprint stimuli.

It could be that, unlike our experience with inverted faces, fingerprint examiners do have experience with inverted prints or, at least, prints that are often not completely upright. Our experience with examiners, however, is consistent with Busey and Vanderkolk [31] in that experts immediately rotate images upright as part of their regular workflow. It could be that the prints were too noisy—and, therefore, too distant from the everyday experience of experts—to reveal an inversion effect. More likely, however, is that the well-established inversion effect seen in *memory* tasks might not hold for *matching* tasks, which makes sense in hindsight. In this experiment, the stimuli to be judged were presented side-by-side, whereas the stimuli in classic inversion effect tasks are spaced in time [44]. Face recognition tasks involve a comparison of instances with those stored in memory, and people rely on the regularities in memory to make judgments. Matching tasks, however, involve a side-by-side comparison and so there is less reliance on memory. It could be that, by virtue of the matching task, there is little chance for an expertise inversion effect to manifest. To the best of our knowledge, there have been no published demonstrations of the inversion effect for faces using a matching task. Therefore, as an avenue for further research, we predict that the classic inversion effect would not be present—or may be heavily attenuated, at least—in a two-alternative, forced choice face matching task, where the two faces are presented side-by-side.

In terms of overall accuracy, experts were more accurate than novices, and experts were just as accurate with similar nonmatching and matching prints, but novices were far less accurate with similar nonmatching prints than with matching prints. The ability of experts to more accurately discriminate similar nonmatching prints replicates the results from previous experiments [22]–[25]. Surprisingly, the absolute level of accuracy for experts was much higher than anticipated. Experts' ability to discriminate similar nonmatches was on par with the results from our previous experiments (e.g., [22]). And the large accuracy difference between experts and novices with similar nonmatches was on par with our previous results as well. These results come despite the fact that visual noise was added to the prints to a point where experts informally reported that there was not enough information present to make an identification. The level of noise was not directly manipulated in this experiment, but the high performance of experts—especially compared to previous experiments—is evidence against the notion that experts engage in careful, deliberate analysis of the minutiae in a fingerprint image in order to make accurate decisions. Indeed, minutiae such as bifurcations, forks, lakes, etc., were not visible in these highly noisy prints. These findings suggest that fingerprint experts are capable of making accurate decisions when the amount of visual information in the prints is decreased—with artificial visual noise, in this case. Future research could examine accuracy as the level of visual noise is systematically manipulated.

## Experiment 2: Prints Spaced in Time

Several avenues of research follow from Experiment 1, but our aim in this program of research is to present various ways of limiting the amount of information available to examiners to better understand the nature of their expertise. When fingerprint examiners compare fingerprints during casework, they usually compare the suspect print and the candidate print on screen at the same time, side-by-side. In Experiment 2, we reduce the amount of information by separating, in time, the two fingerprints that are to be matched. That is, we present the first fingerprint image alone, remove it for a short time, and then present the second fingerprint image alone. In order to perform the task, participants need to hold some information about the first image in memory and use that information to judge whether the second image matches the first. The second image could be a new instance of the print in the first image (target), or the second image could look similar to the first image but be from a different person (distractor). As well as a fingerprint matching task spaced in time, this task can be thought of as a test of short-term memory. We predict that experts will match prints more accurately than novices, especially on similar, nonmatching prints [45].

### Method

#### Participants

xperts were 16 qualified, court-practicing fingerprint experts with experience ranging from 4 to 34 years (*M* = 14.56, *SD* = 7.31) from the Netherlands Forensic Institute and four Australian police organizations: The Australian Federal, New South Wales, Victoria, and Queensland Police. Novices were 42 undergraduates from The University of Queensland who participated for course credit and had no experience with matching fingerprints.

#### Procedure

Participants began by watching a video that explained the experimental task. Part of the video included an example of two fingerprint images, side-by-side, that are of the same finger from the same person, and two fingerprint images, side-by-side, that are from two different fingers. The right side image was the same in both cases (i.e., only the left side image changed). When the experiment began, images of fingerprints were displayed on a computer screen and participants were asked to indicate whether two fingerprints are the same or different (i.e., whether the two fingerprints were of the same finger from the same person, or from two different fingers). The first image appeared on screen for 5 seconds, and a counter on the top left of the screen instructed participants to count up, out loud, from “one” to “five,” to prevent them from verbally encoding the features in the first image. A scrambled visual mask of the first image then appeared for 100 milliseconds followed by the same 5-second counter with no images. A second image then appeared on screen and remained until the participant responded by pressing the “same” or the “different” button. The first image was always a simulated crime scene fingerprint and the second image was always an exemplar. Each participant responded to a total of 36 trials.

#### Stimuli

The stimuli were similar to those from Tangen et al. [23], and consisted of 36 simulated crime-scene prints that were paired with fully rolled exemplar prints. Across participants, each simulated print was paired with a fully rolled print from the same individual (match), and with a nonmatching but similar exemplar (similar distractor). For each participant, each simulated print was randomly allocated to one of the two trial types (target and similar distractor), with the constraint that there were 18 prints in each condition. As described above, matching prints were sourced from the Forensic Informatics Biometric Repository (FIB-R.com), and similar nonmatching prints were obtained by searching the Australian National Automated Fingerprint Identification System.

### Results

**Figure 3:**
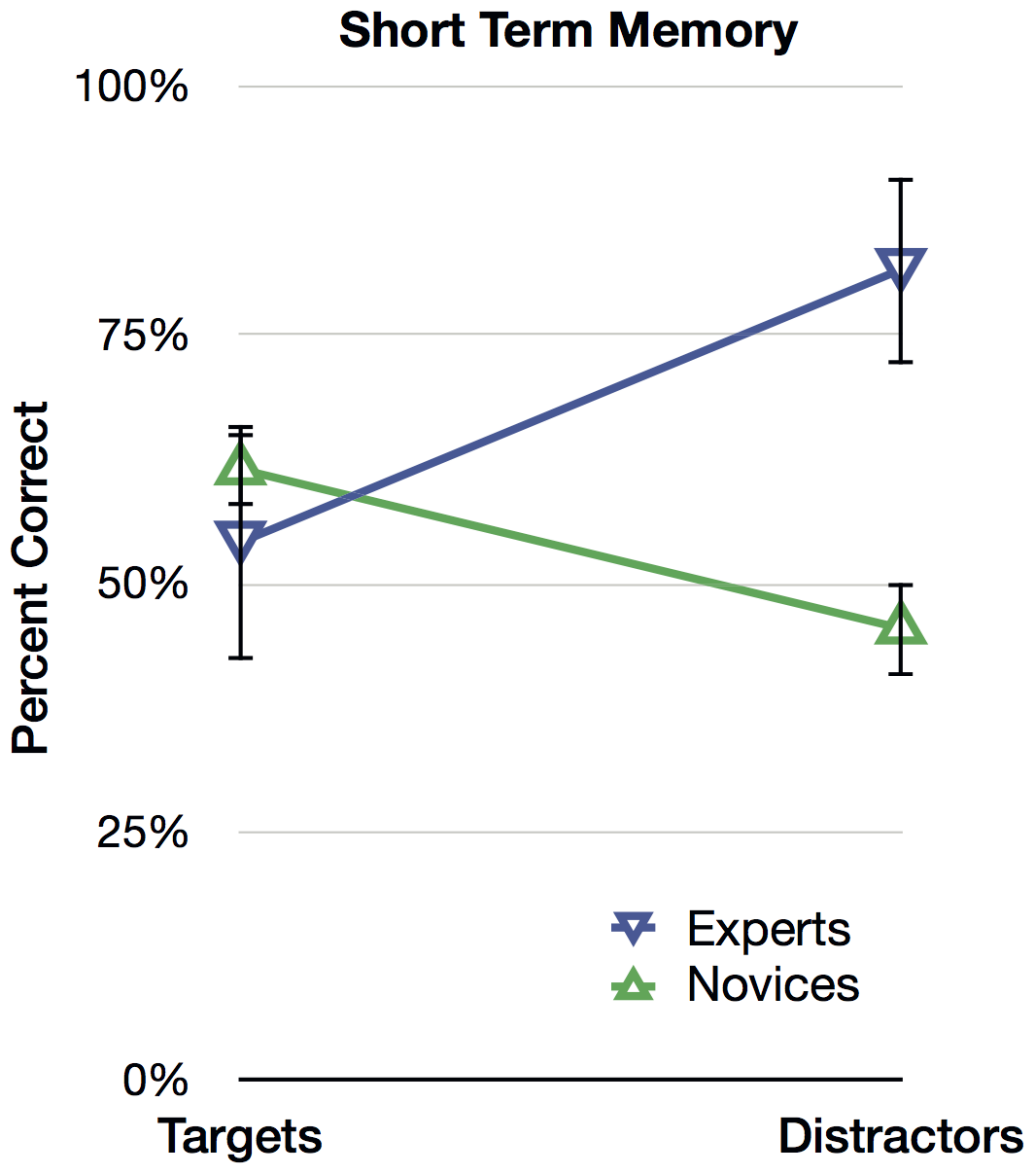
Results from Experiment 2: Prints Spaced in Time. Experts' and novices' mean percentage of correct responses for the two trial types (targets and distractors). Error bars represent 95% within-cell confidence intervals.

Fig. 3 shows the percentage of correct responses (mean recognition accuracy) of experts and novices to target and distractor trials, which were subjected to a 2 (Expertise: expert, novice) ×2 (Trial: target, distractor) mixed ANOVA. Targets and distractors, in this case, can also be thought of as old and new items (in recognition memory parlance), and also as matches and similar nonmatches. Collapsing across Trial to measure the effect of Expertise, experts (67.9%) were more accurate than novices (53.5%), *F*(1, 56)  = 29.455, *p*<.001. Collapsing across Expertise to measure the effect of Trial, people were just as accurate with targets (57.8%) as with distractors (63.5%), *F*(1, 56)  = 2.034, *p* = .159, *ns*. But it is clear from the interaction in Fig. 3 that the absence of a main effect of Trial is driven mostly by experts on distractor trials. The interaction between Expertise and Trial was significant, *F*(1, 56)  = 29.393, *p*<.001, such that experts were more accurate for distractors, and novices were more accurate for targets. As depicted in Fig. 3, expert accuracy was 54.17% for target trials and 81.60% for distractor trials, and novice accuracy was 61.51% for target trials and 45.50% for distractor trials. Experts showed a conservative response bias—they said “Different” on most trials—but their ability to discriminate prints in time was still superior to novices.

### Discussion

We set out to determine the relative performance of experts and novices in a fingerprint matching task where the amount of information was manipulated by separating prints in time. Participants were shown a print, then a five-second blank screen, and then a second fingerprint, and asked to judge whether the two prints were the same or different. For target trials where two prints matched, novices and experts were equally accurate. For distractor trials where two prints did not match but were similar, experts were much more accurate than novices. So, as predicted, we found that experts matched prints more accurately than novices, especially when the prints were similar, but nonmatching. Experts were conservative but still able to discriminate pairs of matching and similar nonmatching prints that were separated by five-seconds. This is an impressive feat given that matching a fingerprint to a memory trace is different from examiners' everyday experience of matching two prints side-by-side. Yet again, as in Experiment 1 and previous research [23], [24], the superior performance of experts seems to be driven largely by an expert's ability to discriminate similar, but nonmatching, prints. That experts had reasonably good memory for prints but novices did not is further evidence for the notion that fingerprint experts can perform accurately with reduced information. This experiment gives a preliminary indication of the short-term memory capacity of fingerprint examiners for domain relevant stimuli. Future research could systematically vary the delay between prints to determine whether short-term memory for meaningful print pairs—such as match or a nonmatch pairs presented side-by-side—is different from short-term memory for less meaningful, individual prints (as in this experiment).

## Experiment 3: Long-term Memory

Having demonstrated that experts show reasonably good short-term memory for prints, we now extend the spacing of prints in time to test the long-term memory of experts. Evans et al. [46] found that expert cytologists and radiologists were no better than novices at recognizing images of objects and scenes, but were better than novices at recognizing images from their domain of expertise. As a further test of the matching ability of fingerprint examiners, and in a further effort to reduce the amount of information available, we separate the prints further in time, thus adding a long-term memory component. That is, participants learn a large set of individual fingerprint images, followed by a five-minute interval, followed by a recognition memory test. (We dub this a “long-term memory task” to differentiate it from the five-second, short-term memory task in Experiment 2, although the boundaries of long and short-term memory delays are somewhat fuzzy [47]). In order to perform the task, participants need to remember whether or not they had seen a print earlier during the learning phase. We predict that experts will match prints (i.e., discriminate old and new items) more accurately than novices, especially on similar, nonmatching prints [45].

### Method

#### Participants

Participants were the same as in Experiment 2 and they completed both experiments in the same session, but there were two fewer novices in this experiment because they failed to complete it. Whether a participant completed Experiment 2 first or Experiment 3 first was counterbalanced.

#### Procedure

Participants were the same as those in Experiment 2. Experiments 2 and 3 were run back-to-back and the order in which expert participants completed the two experiments was counterbalanced. The stimuli between the experiments were entirely independent; none of the prints used in Experiment 2 were used in Experiment 3. Participants watched a video explaining the task. Part of the video included two separate examples of two fingerprint images, side-by-side, that were of the same finger from the same person. In the learning phase, 50 fingerprint images were displayed on screen, one-by-one, for 5 seconds each with a 500 millisecond blank screen between each. Participants were asked to learn the images as best they could and that they would be tested on their ability to recognize the images later.

Following the learning phase, participants completed a word-scramble filler task for five minutes. In order to avoid participants from getting stuck on one word, a 20 second time limit was set so that the correct word would automatically appear in the response field and the participant could move on. Following the filler task, participants watched a second video (30 second duration) explaining that their task was now to recognize the fingerprints that they saw in the learning phase, and that some of the prints they will have seen before and some they will not have seen before. The video reiterated the examples of matching and nonmatching fingerprints. In the test phase, 50 fingerprint images were displayed on screen, one-by-one, with the question, “Have you seen this print before,” and “Yes” and “No” response buttons. Fifty of the fingerprint images were old (i.e., they had been presented in the learning phase) and 50 of the fingerprint images were new (i.e., they had not been presented in the learning phase). The old images in the test phase were not simply the same picture displayed again but, rather, a novel instance of an image of the same finger from the same person (i.e., two “matching” prints are two impressions from the same finger taken at different time). For each participant, the 50 prints from the learning set was randomly chosen from the learning stimuli set of 100.

#### Stimuli

The stimuli consisted of a learning set and a test set. The learning set consisted of 100 photographs of fully rolled individual fingerprint impressions made using a standard elimination pad and a 10-print card. Each card was scanned in color as a 600-dpi lossless Tagged Information File Format (TIFF), cropped to 750×750 pixels, and isolated in the frame. The test set also consisted of 100 photographs of fully rolled individual fingerprint impressions made the same way and they “matched” those from the learning set. That is, each learning print had a corresponding match in the test set, which was a novel photograph instance of the same finger from the same person. In most cases, the two images were inked on two different occasions that were at least two weeks apart. The prints were sourced from the Forensic Informatics Biometric Repository.

### Results

**Figure 4:**
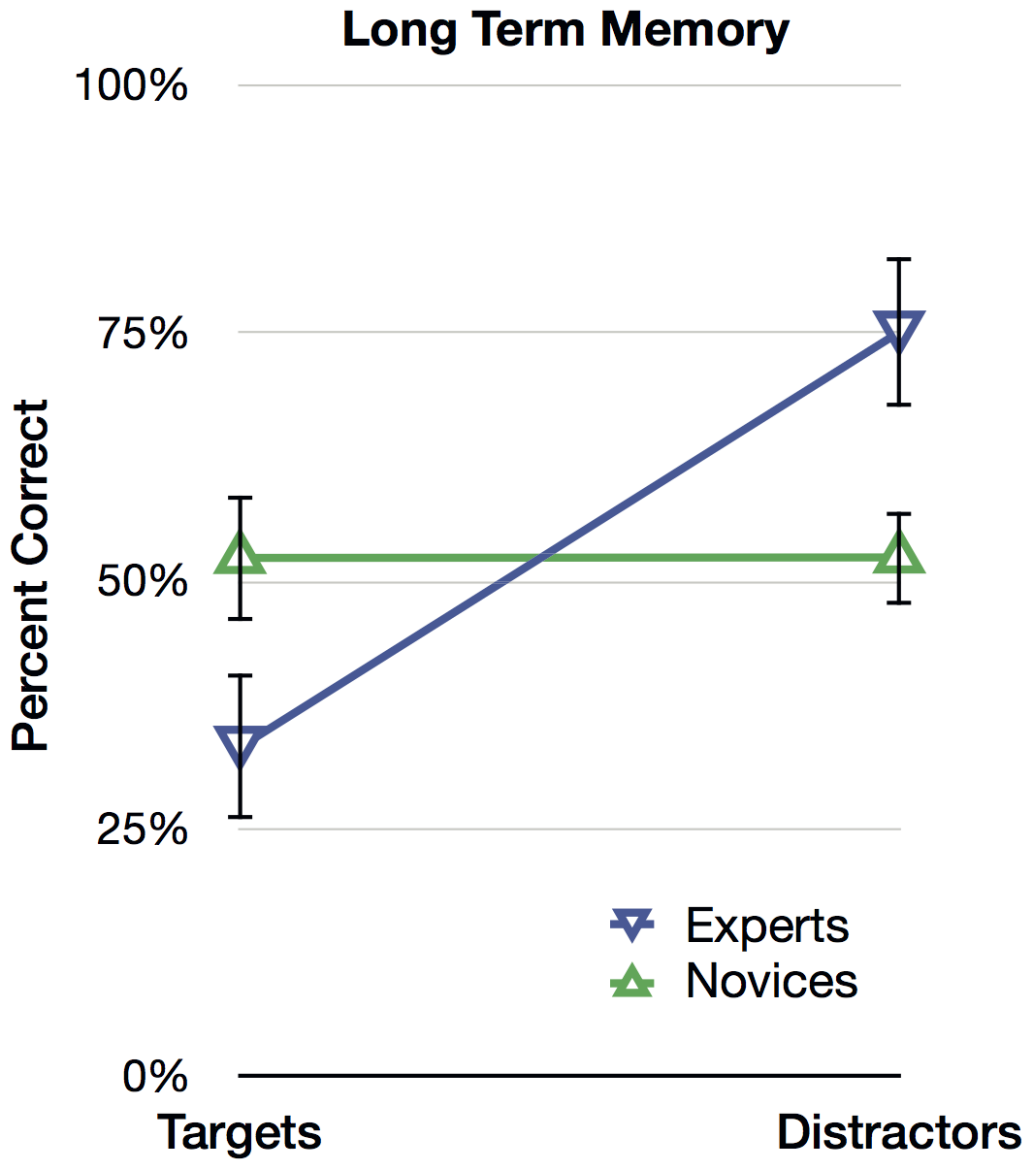
Results from Experiment 3: Long-term Memory. Experts' and novices' mean percentage of correct responses for the two trial types (targets and distractors). Error bars represent 95% within-cell confidence intervals.

Fig. 4 shows the percentage of correct responses (mean recognition accuracy) of experts and novices to target and distractor trials, which were subjected to a 2 (Expertise: expert, novice) ×2 (Trial: target, distractor) mixed ANOVA. Targets and distractors, in this case, can also be thought of as old and new items (in recognition memory parlance), and also as matches and similar nonmatches. Collapsing across Expertise to measure the effect of Trial, everyone was more accurate with distractors (63.8%) than with targets (42.8%), *F*(1, 54)  = 22.971, *p*<.001. But it is clear from Fig. 4 that this main effect is driven by experts on distractors. Collapsing across Trial to measure the effect of Expertise, experts (54.2%) were, unexpectedly, no more accurate than novices (52.4%), *F*(1, 54)  = 2.117, *p* = .151, *ns*. The interaction between Expertise and Trial was significant, *F*(1, 54)  = 22.861, *p*<.001, such that the accuracy difference between targets and distractors for experts (a 41.8% difference) was larger than for novices (a 0% difference). As depicted in Fig. 4, expert accuracy was 33.3% for target trials, and 75.1% for distractor trials, and novice accuracy was 52.4% for target trials and 52.4% for distractor trials. Experts showed a conservative response bias—they said “Different” on most trials—and their overall ability to discriminate prints in long-term memory was no better than novices.

### Discussion

We set out to determine the relative performance of experts and novices in a fingerprint matching task where the amount of information was manipulated by separating the prints by several minutes. Participants were asked to learn 50 fingerprint images and, after a five-minute period, recall those they had seen before from a test set of 100 fingerprints (50 old, 50 new). Overall, long-term recognition memory for experts and novices was the same. Both experts and novices performed around the level of chance. Experts appear to be more conservative than novices, which could be diluting any genuine superior memory ability. Still, on the basis of previous research [46], we expected that experts' long-term memory for prints would be superior to novices. It could be that side-by-side fingerprint matching relies so little on long-term memory that experts do not develop effective mechanisms for encoding and retrieving prints. This experiment gives a preliminary indication of experts' long-term memory for prints (or lack thereof), and suggests that future experiments pushing long-term memory to days, weeks, or months may not prove fruitful. As with Experiment 2, however, future research could test whether long-term memory for meaningful print *pairs* (i.e., match or nonmatch pairs presented simultaneously during learning) is different from memory for less meaningful, individual prints.

## Experiment 4: Short vs. Long Exposure Duration

Having tested the effect of inversion and noise in Experiment 1, short-term memory in Experiment 2, and long-term memory in Experiment 3, we continued to manipulate the information available in fingerprint matching tasks. Examiners suggest that careful, deliberate analysis is the basis of the work that they do [33], [37], but a hallmark of genuine expertise is the ability to accurately perform a domain relevant task quickly and effortlessly [38]. When decisions are made quickly and accurately, it suggests that the underlying cognitive processes are unlikely to be deliberative and analytic. Stimuli for which people have lots of experience, such as natural scenes, can be categorized accurately in around 100 milliseconds [48]–[50], and in as little as 20 milliseconds [51], [52]. People who have a lot of experience with a domain generally perform better than those with little experience, if the regularities of the task are stable enough to learn from.

In Experiment 4, we limit the amount of information available to experts and novices by presenting fingerprint images on screen for only a few seconds. Examiners suggest that careful, deliberate analysis is the basis of the work that they do [33], [37], and so presenting fingerprints quickly should provide little time for them to rely on deliberate, analytic processing. Accurate performance would suggest that experts draw on a repository of prior instances when making judgments about prints. We expect that experts will be more accurate than novices overall. We also expect that experts will be able to discriminate prints presented for two seconds, but that novices will not. Finally, we expect that both novices and experts will be more accurate when viewing pairs of prints for sixty-seconds than when viewing them for two-seconds.

### Method

#### Participants

Two groups participated in the experiment: novices and experts. Novices were 33 undergraduates from The University of Queensland who participated for course credit and had no experience with identifying fingerprints. Experts were 20 qualified, court-practicing fingerprint experts with an average 14 years (*SD* = 8.1) experience from four Australian police organizations: The Australian Federal, New South Wales, Victoria, and Queensland Police.

#### Procedure

We presented participants with pairs of prints displayed side-by-side on a computer screen. Participants were asked to judge whether the prints in each pair matched, using a confidence rating scale ranging from 1 (sure different) to 12 (sure same). Judgments were reported by moving a scroll bar to the left (“different”) or right (“same”). The scale forced a “match” or “no match” decision, where ratings of 1 through 6 indicated “no match,” whereas ratings of 7 through 12 indicated a “match.” The experiment was a mixed 2 (Expertise: expert, novice; between subjects) ×3 (Trial type: match, similar nonmatch, nonsimilar nonmatch; within subjects) ×2 (Deadline: 2 seconds, 60 seconds; within subjects) design. A pair of prints was shown on screen for either 2 seconds or 60 seconds. Specifically, a fixation cross appeared (2 seconds), followed by a scrambled mask of the prints (2 seconds), followed by the prints to be judged (2 seconds or 60 seconds), followed by the same scrambled mask (2 seconds). A slider bar then appeared asking people to rate their confidence from 1 (sure different) to 12 (sure same).

#### Stimuli

Stimuli were the same set of prints we used in Tangen et al. [23], with a simulated crime scene print on the left and a candidate or “suspect” print on the right. There were three types of trials: (1) matches, where the two images were prints of the same finger of the same person, and were separate instances; (2) similar nonmatches, where the two images were prints from two different people but were deemed similar by a database search algorithm; and (3) nonsimilar nonmatches, where the two images were prints from two different people, and the print on the right was randomly selected from the set. There were 36 left side prints in total, and each was paired with a matching print (i.e., a new instance of a print of the same finger from the same person) to create a match trial, or a similar nonmatching print (the result of a national database search as described below) to create a similar nonmatch trial, or a nonsimilar nonmatching print (the right side print was randomly selected from the set of new instance target images). For each participant, each crime scene print was randomly allocated to one of the three trial types, with the constraint that there were 12 prints in each condition, and each pair of prints was randomly assigned to one of the two Deadline conditions. Because of the three trial types, the total possible pairs in the stimulus set was 432 (144×3), but each participant responded to only 144 trials in total. In addition to the three trial types, each trial could be present for either 2 seconds or 60 seconds. Each participant saw 12 matching pairs (6 for 2 seconds and 6 for 60 seconds), 12 similar nonmatching pairs (6 for 2 seconds and 6 for 60 seconds), and 12 nonsimilar nonmatching pairs (6 for 2 seconds and 6 for 60 seconds). Allocation of prints to the Deadline conditions was random, but was counterbalanced by having either all 2 second viewings completed first or all 60-second viewings completed first for each participant.

### Results

**Figure 5:**
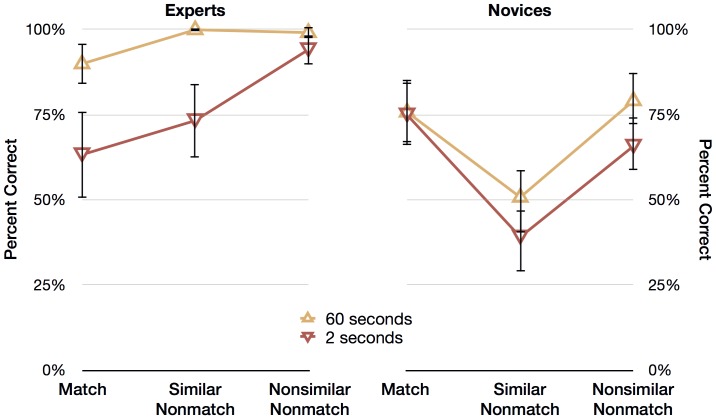
Results from Experiment 4: Short vs Long Exposure Duration. Experts' and novices' mean percentage of correct responses for the three trial types (match, similar nonmatch, and nonsimilar nonmatch) and the two deadlines (60 seconds and 2 seconds). Error bars represent 95% within-cell confidence intervals.

Thirteen novices were randomly selected and removed from the analysis in order to create two groups of equal size: 20 novices and 20 experts. Fig. 5 shows the mean percentage of correct responses of experts (on the left panel) and novices (on the right panel) for the two fingerprint deadlines (2 seconds and 60 seconds) and three trial types (match, similar nonmatch, and nonsimilar nonmatch). We subjected the percentages of correct responses to a 3 (Expertise: expert, novice) ×2 (Deadline: 2 seconds, 60 seconds) ×3 (Trial: target, similar nonmatch, nonsimilar nonmatch) mixed ANOVA. The main effects and interactions that follow are averages collapsed across the twelve experimental conditions, so the percentages described in-text cannot be mapped directly to those depicted in Fig. 2.

#### Main effects

Collapsing across Deadline and Trial, experts were more accurate (86.7% correct) than novices (64.3% correct), resulting in a significant main effect of Expertise, *F*(1, 38)  = 73.343, *p*<.001. Collapsing across Expertise and Trial, people were more accurate when prints were presented for 60 seconds (82.5% correct) compared to 2 seconds (68.5% correct), resulting in a significant main effect of Deadline, *F*(1, 38)  = 42.144, *p*<.001. Collapsing across Deadline and Expertise, although not of particular interest, there was a significant main effect of Trial, *F*(2, 76)  = 9.800, *p*<.001, and follow-up contrasts revealed that nonsimilar nonmatch trials (84.6%) were significantly different from match trials (76.0%), *F*(1, 38)  = 8.01, *p* = .007, and similar nonmatch trials (65.8%) were significantly different from match trials (76.0%), *F*(1, 38)  = 9.21, *p* = .004.

#### Interactions

Of particular interest was the relationship between Deadline and Expertise, because we expected that experts would be better than novices at discriminating prints that are shown for two seconds. It appears from Fig. 5 that experts are responding to the two deadline conditions differently than novices, and there was indeed a significant interaction between Expertise and Deadline, *F*(1, 38)  = 6.284, *p*<.001. The interaction suggests that, although both experts and novices were more accurate with a 60-second deadline, a 60-second deadline increased accuracy for experts (a 19.5% increase) more than it did for novices (a 6.8% increase). As expected, novices were particularly inaccurate with similar nonmatches, and this was borne out by a significant interaction between Trial and Expertise, *F*(2, 76)  = 23.076, *p*<.001, and follow-up comparisons revealing that accuracy on nonsimilar nonmatch trials compared to match trials was greater for experts (a 21.3% increase) than for novices (a 2.9% decrease), *F*(1, 38)  = 14.42, *p* = .001, and accuracy on similar nonmatch trials compared to match trials was greater for experts (an 11.3% increase) than for novices (a 30.4% decrease), *F*(1, 38)  = 36.08, *p*<.001.

The interaction between Deadline and Trial was not significant, *F*(2, 76)  = 1.511, *p* = .227, *ns*, so the effect of deadline did not differ depending on the type of trial. There was a significant interaction between Deadline, Trial, and Expertise, *F*(2, 76)  = 4.596, *p* = .013, suggesting that the interaction between Trial and Expertise differed depending on Deadline. Within-subjects contrasts reveal that experts perform relatively better with short, nonsimilar nonmatch trials compared to novices *F*(1, 38)  = 9.98, *p* = .003 (2 vs. 60 seconds, nonsimilar nonmatch vs. match, expert vs. novice), but the other contrast (2 vs. 60 seconds, similar nonmatch vs. match, expert vs. novice) was not significant, *F*(1, 38)  = .696, *p* = .409, *ns*.

### Discussion

In Experiment 4, we tested whether experts and novices could discriminate matching and nonmatching prints that were presented briefly. We expected that experts would perform well with brief presentations while novices would not. We found that both groups were more accurate when shown prints for 60 seconds than when shown the prints for 2 seconds, and that experts were more accurate than novices overall. Both experts and novices were more accurate with nonsimilar nonmatching and matching prints compared to similar prints, and experts benefitted more from a longer viewing time than did novices. Experts were far more accurate for similar nonmatching prints that were presented for 60 seconds, which replicates results from Tangen et al. [23] and Thompson et al. [24]. Crucially, experts were far more accurate than novices for similar nonmatching prints that were presented for 2 seconds. This difference means that experts could reliably discriminate similar nonmatches even when the prints were presented quickly. This finding is made more interesting by the fact that the vast majority of experts said, informally, that they disliked the experiment because they would not be able to match prints accurately with such a brief presentation—the evidence suggests otherwise.

Experts were more accurate than novices overall, but it appears that their superior performance again lies primarily in discriminating highly similar, but nonmatching prints. Experts were generally more accurate when they had more time to examine the prints, and they benefitted much more from a longer presentation time than did novices. It seems that experts gain more from the kind of analytic processing that is possible with a longer presentation time. It is clear that experts can match prints accurately when there is little time to engage in deliberate reasoning, suggesting that non-analytic processing accounts for a substantial portion of the variance in superior expert accuracy.

## Conclusion

We set out to better understand the nature of expertise in fingerprint identification. In four experiments, we manipulated the amount of “information” available to decision makers in order to characterize the influence of non-analytic cognition. In Experiment 1, we reduced the amount of information available to experts by inverting fingerprint pairs and adding visual noise. There was no evidence for an inversion effect—experts were just as accurate for inverted prints as they were for upright prints—but expert performance with artificially noisy prints was impressive. In Experiment 2, we separated matching and nonmatching print pairs in time. Experts were conservative, but they were still able to discriminate pairs of fingerprints that were separated by five-seconds, even though the task was quite different from their everyday experience. In Experiment 3, we separated the print pairs further in time to test the long-term memory of experts compared to novices. Long-term recognition memory for experts and novices was the same, with both performing around chance. In Experiment 4, we presented pairs of fingerprints quickly to experts and novices in a matching task. Experts were more accurate than novices, particularly for similar nonmatching pairs, and experts were generally more accurate when they had more time.

It is clear that experts can match prints accurately when there is reduced visual information, reduced opportunity for direct comparison, and reduced time to engage in deliberate reasoning. These findings are in stark contrast to the common and consistent claims in formal training, textbooks, and courtroom testimony: fingerprint identification is a ‘scientific process’ that requires careful, thorough analysis in order for judgments to be accurate [33], [36]. We have seen that expert performance is impressive when the amount of information is severely limited. Experts can accurately discriminate matching and nonmatching prints that are artificially noisy, that are spaced in time, and that are seen briefly. We conclude that non-analytic processing plays a key role in expert fingerprint matching. It seems that experts develop a fast and accurate method of fingerprint matching based on their vast experience and familiarity with fingerprints. Emerging evidence supports this instance-based, non-analytic account. For example, Searston, Tangen and Eva [53] demonstrated that both extrinsic information (i.e., crime severity, case familiarity) and intrinsic information (i.e., familiarity of print features) can bias a person's judgment when comparing fingerprints. Pattern recognition is clearly important and accounts for a significant portion of the variance in superior expert performance. Fingerprint experts, through experience, have built up a repository of instances that they draw on to make judgments about novel instances, but it is still not clear how those instances are encoded and stored.

Although non-analytic processing is important for fingerprint matching, these results also indicate that non-analytic processing alone is not sufficient to achieve maximum performance. We found, for example, that experts generally do better when they have a chance to see the prints for a longer duration. It is likely that slow, analytic processing is also important in fingerprint matching, and both kinds of processing will interact, in some way, to drive superior expert performance. Our conclusion that fingerprint identification is largely non-analytic is at odds with general wisdom in fingerprint identification practice and formal training, and at odds with the claims and explanations that are offered in court during expert testimony. The implications are far reaching.

First, if it is the case that vast experience with varied prints is what leads to superior expert performance, then are examiners being trained most effectively? Current fingerprint identification training programs often focus on formal classification and identification rules that date back more than a century [54]. Examiners are trained to classify prints into categories (e.g., loops, arches, and whorls) and use minutiae (e.g., forks, ridge endings, and lakes) to individualize a fingerprint. The findings here, however, suggest that training focused on exposure to many varied instances of matching and nonmatching prints (i.e., to the full range of between and within variation among fingerprints), coupled with accurate and corrective feedback, would be more efficient than training based on formal rules. The training program required to become a qualified expert in an Australian police department is currently five years, minimum. Training focused, at least partly, on the non-analytic basis of fingerprint expertise could conceivably increase the efficiency of training programs without compromising performance standards—we could turn novices into experts more quickly and efficiently. Training ought to be designed to make it easy for examiners to gain a lot of experience with varied prints, and ought to promote a blame-free culture in which people can receive immediate, corrective, and accurate feedback about their performance. The best way to design a system that promotes the accumulation of a multitude of instances with corrective feedback remains an open question.

Second, given that much of the decision making process of fingerprint experts is non-analytic, should experts have to justify the basis of their decisions in court? Expertise in a domain does not necessarily include the ability to articulate the reasoning behind judgments and decisions [55]–[58]. The information that fingerprint examiners think, or retrospectively report, that they rely on may bear limited resemblance to the information that they actually use. That is, while examiners can accurately tell us whether two fingerprints match or not, it is unlikely that they can accurately tell us *why* they think two fingerprints match or not. This notion is in contrast to legal practice and assumptions about expertise. In their admissibility standards for expert opinion evidence, many jurisdictions specify the need for “specialized knowledge” for expert opinion evidence. If the judgments of examiners are not readily articulable, then courts should not expect accurate answers when they ask experts to introspect about the basis of their decision in the particular case, or the nature of their expertise in general—an expert's answer could be inaccurate at best and misleading at worst. Edmond, Thompson, and Tangen [59] have proposed an alternative model of testimony that circumvents this issue, and that is capable of incorporating emerging empirical evidence on the nature of expertise in fingerprint identification of the kind presented here.
